# 3D-Printed Multi-Stimulus-Responsive Hydrogels: Fabrication and Characterization

**DOI:** 10.3390/mi16070788

**Published:** 2025-07-01

**Authors:** Jinzhe Wu, Zhiyuan Ma, Qianqian Tang, Runhuai Yang

**Affiliations:** 1 School of Electronic Engineering, Naval University of Engineering, Wuhan 430033, China; wujinzhe@ustc.edu (J.W.); zhiyuan_ma@nue.edu.cn (Z.M.); 2 Chaohu Clinical Medical College, Anhui Medical University, Chaohu 238000, China; qqt19960806@163.com; 3 The Chaohu Hospital of Anhui Medical University, Chaohu 238000, China

**Keywords:** stimulus-responsive hydrogels, direct ink writing, wearable flexible sensors

## Abstract

Stimulus-responsive hydrogels have broad applications in the biomedical, sensing, and actuation fields. However, conventional fabrication methods are often limited to 2D structures, hindering the creation of complex, personalized 3D hydrogel architectures. Furthermore, hydrogels responding to only a single stimulus and delays in fabrication techniques restrict their practical utility in biomedicine. In this study, we developed two novel multi-stimuli-responsive hydrogels (based on Gelatin/Sodium Alginate and Tannic Acid/EDTA-FeNa complexes) specifically designed for direct ink writing (DIW) 3D printing. We systematically characterized the printed properties and optimized component ratio, revealing sufficient mechanical strength (e.g., tensile modulus: Gel/SA-TA ≥ 0.22854 ± 0.021 MPa and Gel/SA-TA@Fe^3+^ ≥ 0.35881 ± 0.021 MPa), high water content (e.g., water absorption rate Gel/SA-TA ≥ 70.21% ± 1.5% and Gel/SA-TA@Fe^3+^ ≥ 64.86% ± 1.28%), excellent biocompatibility (e.g., cell viability: Gel/SA-TA and Gel/SA-TA@Fe^3+^ ≥ 90%) and good shape memory performance (e.g., the highest shape recovery rate of Gel/SA-TA reaches 74.85% ± 4.776%). Furthermore, we explored electrical characteristics, showing that the impedance value of Gel/SA-TA@Fe^3+^ hydrogel changes significantly under finger bending and NIR irradiation. This investigation demonstrates the potential of these 3D-printed multi-stimuli hydrogels for applications such as wearable flexible strain sensors.

## 1. Introduction

With the development of human–computer interaction [1,2], augmented reality devices [3,4], and electronic skin technologies [5,6], traditional solid sensors can no longer meet certain requirements. Flexible sensors have attracted extensive attention due to their enhanced performance, such as their tensile strength, performance stability, and cell compatibility [7,8,9,10]. Furthermore, flexible sensors are easier to bend and attach to bioplanes [11,12]. Smart multi-responsive hydrogels are ideal materials for designing flexible sensors [13,14,15]. Compared with other materials, such as liquid crystals and polymer films, these hydrogels have many advantages: a morphology adjustable enough to respond to a variety of external stimuli (temperature, humidity, pH, ions, etc.), excellent bio-compatibility, and degradability [16,17].

However, fabricating smart multi-responsive hydrogel 3D structures with sufficient mechanical properties remains challenging. Compared with traditional processing methods such as fixture fixation [18], hydrogel masks [19], and extrusion [20], additive manufacturing (AM) technology has received tremendous attention because it can provide customized geometry and sufficient mechanical strength while preserving flexibility in microstructural control [21,22]. DIW is an AM technology based on ink extrusion, where the ink is extruded from a micro-nozzle and deposited into a custom pattern under the movement of the printing substrate [23]. Compared with other AM technologies, DIW has a wide range of printable materials, is simple to operate, and is capable of rapid prototyping, all of which contribute to its great potential for manufacturing multi-scale and multifunctional structures [24,25,26].

Unfortunately, the properties of conventional smart multi-responsive hydrogels cannot support DIW printing [27,28]. Ideal DIW bioink depends on three factors: (a) printability, (b) shape fidelity, and (c) bio-compatibility [29]. Various types of smart multi-responsive hydrogels have been designed [30,31,32], Of these, gelatin is commonly used as a precursor material to prepare smart multi-responsive hydrogels due to its excellent biocompatibility and unique triple helix structure network; this structure can provide sufficient conditions to alter the permanent or temporary shape of a hydrogel [33,34]. In addition, sodium alginate (SA) is often added to improve mechanical strength and regulate the rheological properties of gelatin hydrogels [35,36]. Despite this, the mechanical properties of hydrogels still create difficulties when aiming to obtain 3D structures. To improve structural strength, tannic acid (TA) has been added to hydrogel systems as a phenolic crosslinking agent [37,38].

Moreover, recent advancements have increasingly focused on what kind of non-invasive external stimulus should be incorporated into hydrogel designs, such as light, heat, electricity, magnetism, or ultrasound [39,40,41,42]. Compared with other non-invasive external stimuli, near-infrared (NIR) light (electromagnetic waves with wavelengths between 780 nm and 2526 nm) has strong tissue penetration, causes little light damage, and has low spectral interference. It is common for NIR stimulus hydrogels to disperse infrared light stimulus–response materials in the shape memory material matrixes based on temperature sensitivity characteristics [43,44]. Usually, graphene oxide, carbon nanotubes, or carbon nanoparticles enable the hydrogel system to achieve a photothermal effect, but these additions are not biocompatible. Therefore, Fe^3+^ ions have also been introduced into a hydrogel system [45,46,47]. Sodium iron ethylene diamine tetra acetic acid (sodium iron ethylene diamine tetra acetic acid [EDTA]) has also been used as an Fe^3+^ ion carrier [48]. Although such a composite hydrogel system can theoretically overcome many problems, including poor printability, insufficient mechanical strength, and low shape memory efficiency, the specific ratio concentration of the composite hydrogel system still requires continuous optimization and systematic study.

In this study, a kind of Gel/SA-TA bioink was designed for DIW printing. Then, the properties of this bioink were characterized under different component ratios; namely, its mechanical properties, micro-morphology, water content, swelling performance, shape memory performance, and biocompatibility. On this basis, Fe^3+^ was introduced into a Gel/SA-TA hydrogel, a Gel/SA-TA@Fe^3+^ bioink was designed, and its component ratio was optimized. Finally, to verify the possibility of using Gel/SA-TA@Fe^3+^ bioink to prepare flexible sensors, the ink’s NIR and deformation stimulation electrical response performance were characterized.

## 2. Materials and Methods

### 2.1. Preparation of Gel/SA-TA Bioinks

**Figure 1:**
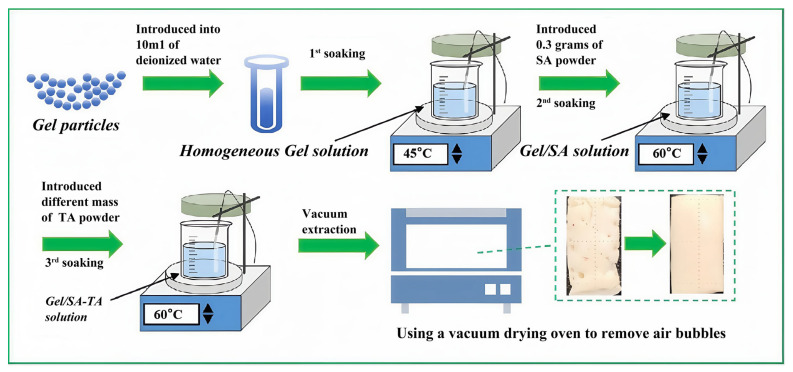
Schematic diagram of the preparation process.

A schematic diagram of the preparation process is shown in Figure 1. Initially, gel particles of a designated mass were introduced into 10 mL of deionized water, followed by magnetic stirring within a thermostatic water bath at a temperature of 45 °C until a homogeneous gel solution was achieved. Subsequently, 0.3 g of SA powder was incorporated into 10 mL of the gel solution, maintained at a temperature of 60 °C, and subjected to magnetic stirring for 2 h. Ultimately, TA powder was integrated into the resultant mixture, with further magnetic stirring at 60 °C for an additional 2 h to yield a consistent ink composition. Lastly, the cylinder was evacuated at room temperature using a vacuum-drying oven to remove air bubbles.

Based on our previous studies [49], bioinks composed of Gel/SA and Gel/SA-TA with varying constituent proportions were formulated, with the precise ratios detailed in Table 1.

### 2.2. Characterization of Gel/SA-TA Bioinks

Fourier transform infrared spectroscopy: FTIR spectroscopy is a well-known technique for characterizing the chemical structure of hydrogels, with high specificity and sensitivity in detecting the vibrational bonds of functional groups, identifying characteristic absorption peaks, and tracking changes in chemical bonds before and after reactions [50,51]. A Fourier infrared imaging spectrometer (NicoletIS50, Nicolet Company, Madison, WI, USA) was used to characterize the chemical bond differences before and after different constituent proportions of Gel/SA-TA hydrogel samples were prepared to verify whether the functional groups between the hydrogel and TA were compatible. Firstly, the samples were dried to prevent the absorption of H_2_O from affecting the result. Then, the KBr and samples were ground with a mass ratio of 100:1 and pressed into a uniform transparent sheet. Finally, we put the samples in a sample tank for scanning and exported the data.

Characterization of 3D printing performance: The consistency of bioink is crucial for the DIW 3D printing process [52]. This investigation assessed the viscosity of Gel/SA bioink across varying gel concentrations at an ambient temperature (25 °C). The objective was to ascertain the ink’s 3D printing capabilities and identify the ideal gel concentration for the Gel/SA ink formulation. Additionally, for the viscosity of the Gel/SA-TA hydrogel, varying TA concentrations at diverse mixing ratios were determined using a rotational rheometer (NETZSCH Kinexus Lab+, NetCH, Iserlohn, Germany). The tests were conducted at a constant temperature of 25 °C, an atmospheric pressure of 3bar, and a shear rate spanning from 0.1 to 100 s^−1^.

### 2.3. Three-Dimensional Printing Gel/SA-TA Bioink

Firstly, a 3D model was constructed using Blender (NaN Company, Amsterdam, The Netherlands), the model was imported into Ultimaker Cura (Ultimaker Company, Geldermalsen, The Netherlands), and the printing parameters of the Gel/SA-TA bioink were set. The filling pattern was a grid, the filling density was 40%, the height of each layer was 0.3 mm, and the printing temperature of the cylinder was 25 °C. The printing speed was 8 mm/s. A needle with an inner diameter of 0.42 mm was used to print the Gel/SA-TA bioink at room temperature (25 °C). The composition of the DIW 3D printer is shown in Figure S1.

### 2.4. Characterization of Gel/SA-TA Bioink Printing Samples

**Mechanical property testing:** Good tensile strength is necessary to produce suitable stimulus-responsive hydrogels for biomedical applications [13]. Many studies have relied on tensile experimental data to demonstrate the possibility of hydrogel fabrication in devices [53,54]. In this study, different proportions of Gel/SA-TA bioink were printed into 10 mm × 20 mm × 1 mm cuboid scaffolds using a DIW 3D printer. Then, an electronic universal testing machine (1 kN; RGM30, REGER company, Qingdao, China) was used to conduct tensile tests on the scaffolds at a tensile speed of 2mm/min until the samples were broken. Each group of samples was tested three times to reduce experimental error, and the final results were averaged. The sample load is denoted as F_o_, the width is denoted as W_o_, the thickness is denoted as H_o_, the length before the tensile test is denoted as L_o_, and the extension length is denoted as L_1_. The stress and strain of each scaffold can be calculated using Formulas (1) and (2):

(1) $$\sigma (MPa)=F_{o}/(W_{o}\times H_{o}),$$

(2) $$\varepsilon =L_{1}/L_{o},$$

**Observation of micro-morphology:** In biomedical applications, the adhesion, proliferation, and diffusion of cells are extremely dependent on the microstructure of composite functional hydrogels, and the swelling and mechanical properties of hydrogels are closely related to their microstructure [55]. In this study, the prepared Gel/SA-TA bioink was first printed into a 20 mm × 10 mm × 5 mm cuboid using a DIW 3D printer and crosslinked with a 4 wt% CaCl_2_ solution. The Gel/SA-TA hydrogel strip then rested at −40 °C for 12 h. Subsequently, it was placed in a −80 °C environment for 24 h, and finally, it was placed in a lyophilizer for 48 h. The freeze-dried hydrogel was then removed, and the cross-section was exposed using external force. Finally, the microscopic morphology of the hydrogel was observed under a field emission scanning electron microscope (NOVA NANA230, FEI Company, Hillsboro, OR, USA).

**Water content test:** Water content and swelling performance are important criteria for characterizing the performance of hydrogels, such as nutrient penetration and exchange and oxygen transfer [56]. In this study, the prepared Gel/SA-TA bioink was printed into a 10 mm × 10 mm × 5 mm cuboid using a DIW 3D printer, crosslinked with a 4 wt% CaCl_2_ solution, and the m_0_ was weighed. Then, the prepared Gel/SA-TA hydrogel strip was quick-frozen at −80 °C for 1 h. Subsequently, it was placed in a lyophilizer and vacuum dried for 36 h until the sample mass was constant, and the m_1_ was weighed. Three parallel samples were set for each group. The water content, α, of each Gel/SA-TA hydrogel can be calculated using Formula (3):

(3) $$\alpha (\%)=(m_{0}-m_{1})/m_{0}\times 100\%,$$

**Swelling performance test:** The prepared Gel/SA-TA bioink was printed into cylinders with a 10 mm diameter and a 10mm height using a DIW 3D printer, which was crosslinked using a 4 wt% CaCl_2_ solution. Then, the gel samples were immersed in PBS (pH = 7.4) solution at 37 °C until the mass was constant, and the W_0_ was weighed. The swelling Gel/SA-TA hydrogel strips were then placed in a −80 °C environment for 1 h and then placed in a lyophilizer for 36 h. The W_1_ was then weighed. Three parallel samples were set for each group. The swelling rate, β, of each Gel/SA-TA composite functional hydrogel can be calculated using Formula (4):

(4) $$\beta (\%)=(W_{0}-W_{1})/W_{0}\times 100\%,$$

**Shape memory performance characterization:** It is critical that the temporary shape of memory gel materials can stabilize and that the original shape can be completely recovered under stimulation. In this study, the prepared Gel/SA-TA bioink was printed as a standard tensile spline using a DIW 3D printer and crosslinked using a 4 wt% CaCl_2_ solution. The original length of the sample was recorded as D_0_. The prepared hydrogel samples were placed in a 50 °C environment for 60 s and then placed in a room temperature environment for 30 s. The linear length of both ends of the sample after shape recovery was denoted as D_1_. Three parallel samples were set for each group. The shape recovery rate, γ, of each Gel/SA-TA composite functional hydrogel can be calculated using Formula (5):

(5) $$\gamma (\%)=(D_{0}-D_{1})/D_{0}\times 100\%,$$

**Biocompatibility characterization:** Cytocompatibility is an indispensable index for evaluating biomedical materials. 293T cells are recommended as a standard model for biocompatibility screening in international standards (such as ISO 10993-5 [57]), are highly sensitive to toxicity, and are widely used for initial safety assessments of hydrogels [55,56]. Hence, in this study, human renal epithelial cells (293T) were first cultured in DMEM containing 10% fetal bovine serum. Cells were seeded in polystyrene tissue culture dishes and cultured in a 5% CO_2_ cell incubator at 37 °C. The medium was changed daily, and the cells were separated with trypsin–EDTA every 4 days and passaged at a 1:3 ratio. The prepared Gel/SA-TA bio-co-culture pre-ink was then printed as a 10 mm × 10 mm × 5 mm cuboid using a DIW 3D printer. The printed scaffolds were immersed in 75% ethanol, completely sterilized under UV light for 1 h, washed with phosphate-buffered saline 3 times, and incubated in a 24-well plate containing DMEM overnight. The cell suspension was then seeded on the scaffolds at a volume of 2 × 10^5^ cells/scaffolds in 1ml of DMEM (1 scaffold was placed in each well of the 24-well plate). The cell-seeded scaffolds were incubated at 37 °C at 5% CO_2_, and the medium was changed every other day.

### 2.5. Preparation of Gel/SA-TA@Fe^3+^ Bioinks

EDTA-FeNa powder was added to the Gel/ SA-TA shape memory hydrogel (gel concentration was 20 wt%, SA concentration was 3 wt%, and TA concentration was 5 wt% and 6 wt%) after optimizing the composition ratio, and the powder was magnetically stirred at 60 °C for 2 h until a uniform shear thinning ink was formed. The barrel was subsequently evacuated at room temperature using a vacuum-drying oven to remove air bubbles. The above steps were performed more than three times; when the ink in the cylinder no longer produced bubbles, bubble removal was complete. Based on our previous study [49], Gel/SA-TA@Fe^3+^ bioinks with different component ratios were prepared, and the specific component ratios are shown in Table 2.

### 2.6. Characterization of Gel/SA-TA@Fe^3+^ Bioink Printing Samples

Using the Gel/SA-TA material scaffold 3D printing method, a Gel/SA-TA@Fe^3+^ scaffold was prepared. The same test method was used to analyze the properties of the Gel/SA-TA@Fe^3+^ materials and scaffolds, namely, the 3D printing properties, mechanical properties, microstructure, water content, swelling properties, and biocompatibility. In addition, since Gel/SA-TA@Fe^3+^ has photothermal properties, the photothermal properties of the Gel/SA-TA@Fe^3+^ were also tested.

**Photothermal test:** Hand-held thermography (HM-TPK20-3AQF/W) was used to characterize the photothermal properties of the Gel/SA-TA@Fe^3+^ hydrogel with different Fe^3+^ and TA concentrations. In total, 1.3 mL of Gel/SA-TA@Fe^3+^ bioink with different EDTA-Fe^3+^ and TA concentrations was placed in a 1.5 mL centrifuge tube, which was irradiated using a near-infrared pulse laser with a wavelength of 808nm and an intensity of 0.2 W/cm^2^ for 10 min. The temperature changes in the centrifuge tube during this period were recorded using a thermal imager.

### 2.7. Measurement of Gel/SA-TA@Fe^3+^ Impedance Feedback Signals in Response to Deformable and Infrared Stimuli

Given the excellent tensile and NIR stimulation response properties of the Gel/SA-TA@Fe^3+^ hydrogel, its conductivity under bending and NIR irradiation was characterized. 

**Measurement of deformation stimulation impedance feedback signal:** The two ends of the prepared Gel/SA-TA@Fe^3+^ hydrogel sample adhered to an appropriate length of conductive glue, and the sample was connected to an electrochemical workstation. Then, the bending of the hydrogel was adjusted and recorded, and a finger bending–straightening cycle was applied every 20 s; the total measurement time was 200 s. The bending angle of the hydrogel was tested in a 30° group, a 60° group, and a 90° group. The measurement methods and parameters of each group referred to the above steps.

**Infrared stimulation impedance feedback signal measurement:** The sample was connected to the electrochemical workstation using the same method. Then, the IMPT impedance–time test method was applied. An NIR switch cycle was applied every 20 s, with a total measurement time of 120 s. The infrared power density values were tested in a 0.28 W/cm^2^ group, a 0.4 W/cm^2^ group, and a 0.56 W/cm^2^ group. The measurement methods and parameters of each group referred to the above steps.

## 3. Results and Discussion

### 3.1. Performance of Gel/SA-TA Bioprinting Ink

**Figure 2:**
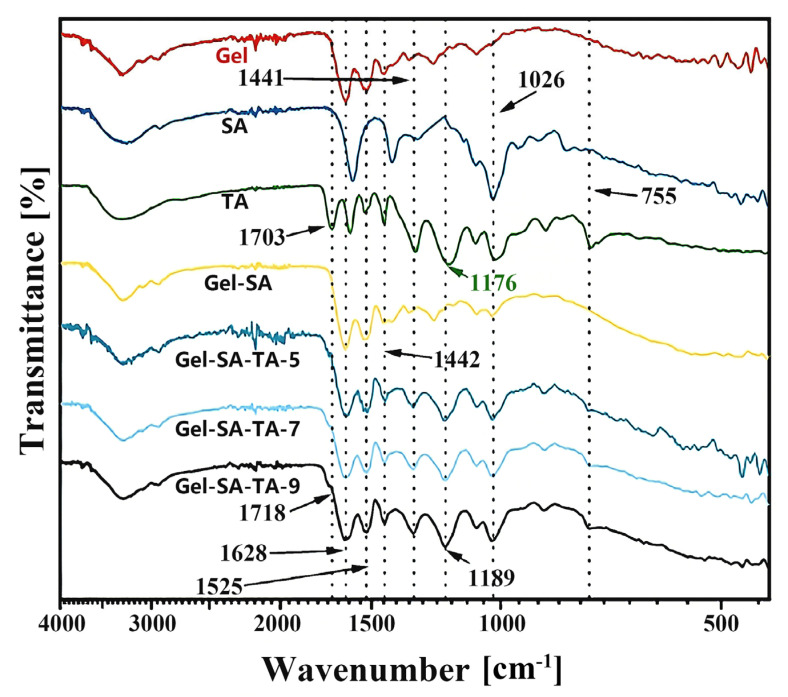
FTIR spectra of gel, SA, TA, and different proportions of lyophilized gels.

**Analysis of Fourier transform infrared spectroscopy results:** Figure 2 shows the FTIR spectra of the freeze-dried Gel, SA, and TA samples and the gel obtained by mixing them in different ratios. In the infrared spectrum of Gel/SA, the strong absorption peak at 1692 cm^−1^ was due to C=O stretching vibration in the amide structure (amide I band); the signal at 1525 cm^−1^ was from N-H bending vibration (amide II band); and the peak at 1442 cm^−1^ was from C-H asymmetric bending vibration. In addition, the weak peak near 1026 cm^−1^ comes from the C-O stretching vibration in the SA component. However, a new peak at 1718 cm^−1^ can be attributed to the C=O stretching vibration that appeared in the lyophilized gel after adding TA. This peak is derived from the ester group structure of TA. Compared with the sample without TA (Gel/SA), the infrared spectrum of the sample with TA significantly broadened at 1628 cm^−1^. This is because the C=C stretching vibration of the benzene ring structure in the TA molecule is located at 1607 cm^−1^, and this peak is superimposed with the esterified peak at 1628 cm^−1^, broadening the signal. As the amount of TA added increased, the signal widened. However, the signal at 1525 cm^−1^ was due to N-H bending vibration. The peak at 1442 cm^−1^ can be assigned to C-H asymmetric bending vibration. Notably, a new peak at 1189 cm^−1^, owing to C-O asymmetric stretching vibration, appeared after the addition of TA. In TA, this peak is located at 1176 cm^−1^. An obvious blue shift occurs at this peak, related to the breaking of old hydrogen bonds and the formation of new hydrogen bonds after the formation of the gel. Similarly, after gel formation, the signal attributed to the esterified C=O stretching vibration at 1703 cm^−1^ also red-shifted to 1718 cm^−1^, confirming the formation of a hydrogen bond interaction between the TA and Gel/SA matrix. The peak was more obvious in the samples with more TA added.

In addition, the signal at 1026 cm^−1^ is due to C-O symmetric stretching vibration. Compared with the pure Gel/SA hydrogel, this peak appeared in the lyophilized gel after adding TA, which is the characteristic signal of structures such as the phenolic hydroxyl and ester groups in TA. However, the broad absorption at 755 cm^−1^ is the characteristic signal of C-H out-of-plane bending vibration in the benzene ring structure, which comes from the aromatic structure of TA. These characteristic peak changes in the samples before and after adding TA may indicate an interaction between the functional groups of the biopolymer and those of TA; thus, the components have good compatibility.

**Figure 3:**
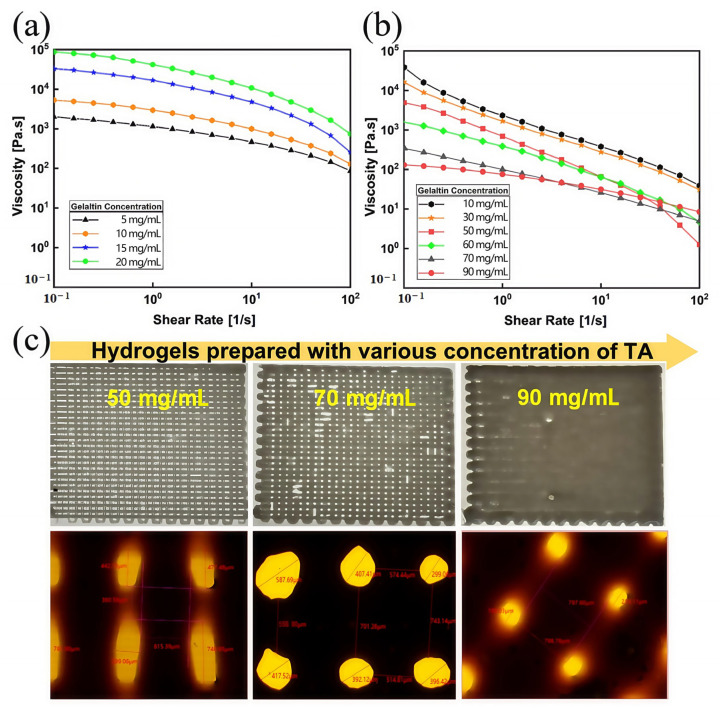
The 3D printing performance characterization results. (**a**) Rheological properties of Gel/SA bioinks at room temperature (25 °C) at different gel concentrations; (**b**) rheological properties of Gel/SA-TA bioink at room temperature at different TA concentrations; (**c**) printed renderings of Gel/SA-TA bioink with different TA concentrations at room temperature.

**Analysis of 3D printing performance characterization results:** As shown in Figure 3a, when the gel concentration increased from 5 wt% to 20 wt%, the viscosity of the Gel/SA bioink increased. At the same time, the viscosity of each bioink group significantly decreased when the shear rate increased, indicating that Gel/SA ink had excellent shear-thinning characteristics. However, when the gel content reaches 25 wt%, it is difficult to completely remove the bubbles from Gel/SA ink. Considering printing resolution and efficiency, a 20 wt% gel concentration was selected as the Gel/SA bioink component in this study. At this concentration at room temperature, the viscosity of the Gel/SA bioink was about 1 × 10^5^. The material viscosity was too great, which can easily block the printing needle. TA was selected as the rheological modifier for Gel/SA to make the material more suitable for the DIW process. Therefore, we tested the viscosity of Gel/SA-TA bioinks with different TA concentrations at room temperature. As shown in Figure 3b, when the shear rate increased from 0.1 to 100 s^−1^, the Gel/SA-TA viscosity was significantly reduced to less than 10^2^ Pa·s, indicating that the Gel/SA-TA bioink had excellent shear-thinning characteristics. The viscosity of the Gel/SA-TA bioink increased with higher TA concentration, and the result may be due to the intermolecular forces between the TA and Gel/SA hydrogel matrix.

At the same time, the effect of printing the same mesh model with Gel/SA-TA bioink with different TA concentrations was also compared, as shown in Figure 3b,c. When the TA concentrations were 5 wt%, 7 wt%, and 9 wt%, the average pore diameters of the grid were 490.055 μm, 416.703 μm, and 207.7 μm, and the average pore interval lengths were 380.59 μm, 665.067 μm, and 798.69 μm. This indicates that introducing TA from 5 wt% to 7 wt% improves the 3D printing performance of the Gel/SA material.

### 3.2. Performance of Gel/SA-TA Bioink Printing Samples

**Figure 4:**
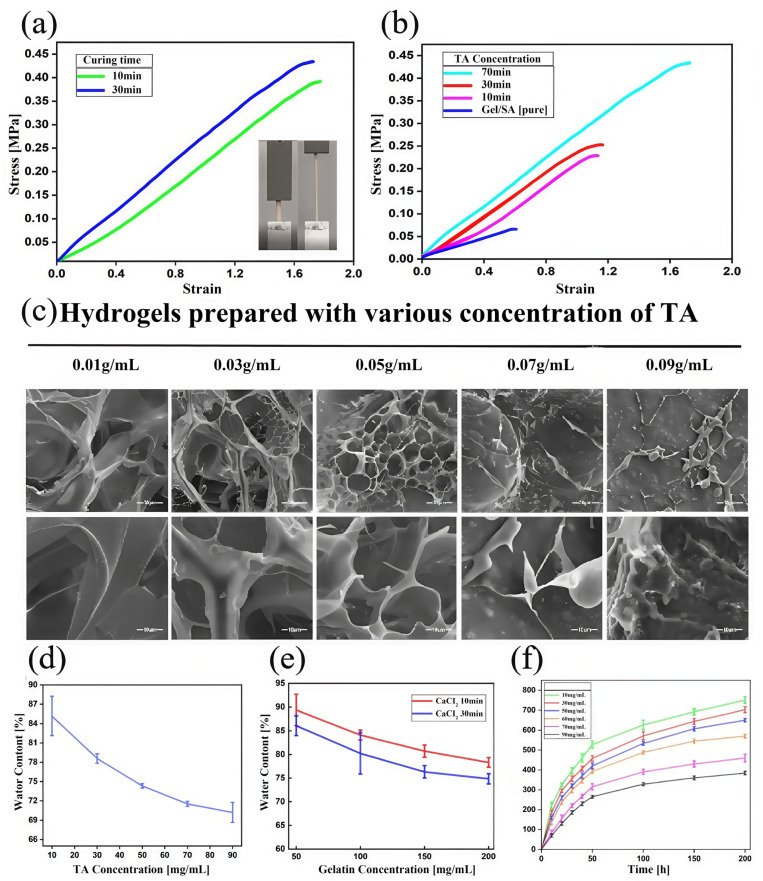
The physical properties of Gel/SA-TA bioink printing samples. (**a**) Gel/SA-TA hydrogel stretching curves at different crosslinking times; (**b**) Gel/SA-TA hydrogel stretching curves at different TA concentrations; (**c**) electron microscopic scan of Gel/SA-TA hydrogel morphology at different TA concentrations (the scale is 10 μm); (**d**) water content curve of Gel/SA-TA hydrogel under different TA concentrations after crosslinking for 30 min in CaCl_2_ solution; (**e**) water content curves of Gel/SA-TA hydrogels with different gel concentrations at a TA concentration of 5wt% using different CaCl_2_ solution crosslinking times; (**f**) swelling rate curves of Gel/SA-TA hydrogel at different TA concentrations. All experiments were performed in triplicate, and data are reported as mean ± SD (n = 3).

**Analysis of mechanical properties:** As shown in Figure 4a, with the increase in TA concentrations, the average tensile modulus of the Gel/SA-TA spline increased from 0.06614 ± 0.003 MPa to 0.22854 ± 0.021 MPa. When the TA concentration was 7 wt%, the average tensile modulus was 0.434 ± 0.035 MPa, which was about 6.6 times higher than that of the Gel/SA hydrogel. This is because the number of intermolecular covalent bonds between the TA and Gel/SA hydrogel matrix increased with the increase in tannic acid concentrations, confirming the rationality of our material design. In addition, we also tested the tensile properties of Gel/SA-TA hydrogel prepared using different CaCl_2_ solution (4 wt%) crosslinking times. As shown in Figure 4b, when crosslinked for 30 min, the average tensile modulus was approximately 1.1 times greater than that of hydrogels crosslinked for only 10 min.

**Analysis of microscopic morphology:** As shown in Figure 4c, the Gel/SA-TA hydrogel developed an interconnected porous structure when the TA concentration was below 7 wt%. Specifically, those with 1 wt% and 3 wt% TA formed large microscopic pores, averaging between 73 and 152 μm. As the TA concentration increased, its microstructure transitioned into smaller honeycomb pores, reaching a minimum diameter of about 16 μm. At higher TA concentrations, the hydrogel developed a non-typical, highly compact pore structure due to intermolecular covalent bond formation between the TA and Gel/SA groups. This increased TA concentration also resulted in a more compact hydrogel structure overall.

**Analysis of water content performance:** Figure 4d,e show that the water content of Gel/SA-TA hydrogel decreases with increasing gel concentrations, TA concentrations, and CaCl_2_ solution crosslinking times. This indicates stronger interactions between TA and the Gel/SA matrix under these conditions. The lowest water content was 70.21% ± 1.5%. Figure 4f shows the swelling curve of this hydrogel at different TA concentrations after 30 min of crosslinking. Each group reached near-swelling equilibrium after 200 h of immersion in PBS, with the highest swelling degree (750.6% ± 17.6%) occurring at 1 wt% TA. The swelling performance decreased as the TA concentration increased, consistent with the water content results. Higher TA concentrations increase the hydrogel’s crosslinking density, reducing water absorption and the swelling rate.

**Figure 5:**
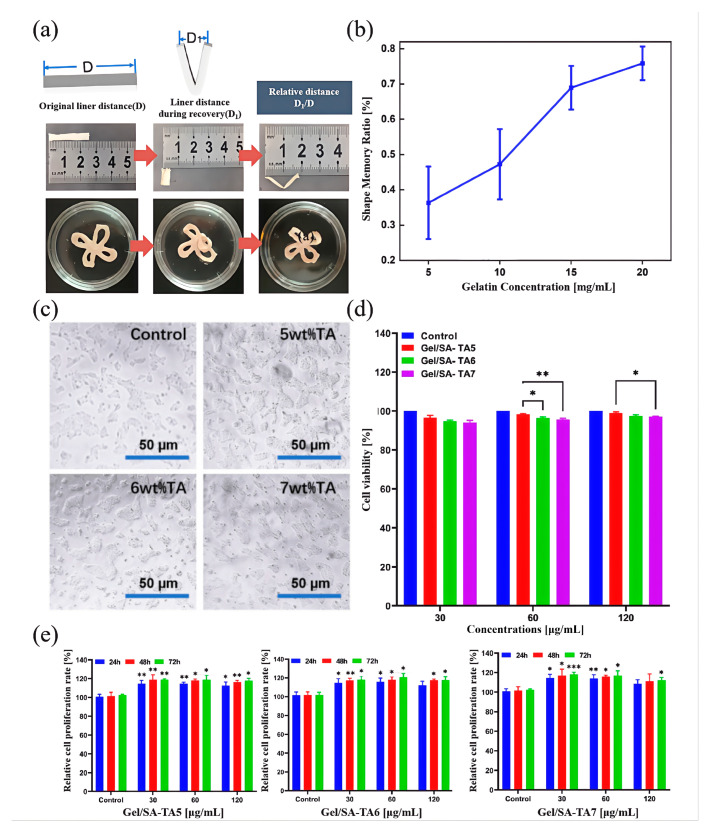
Shape memory performance and biocompatibility of Gel/SA-TA bioink printing samples. (**a**,**b**) Schematic diagram of hydrogel shape memory recovery rate of the relative distance, D_1_/D; (**c**) inverted microscope image of 293T cells after Gel/SA-TA hydrogel coculture for 72 h; (**d**) analysis of cell activity of Gel/SA-TA hydrogel with different TA concentrations in co-culture with 293T cells at different concentrations—data are reported as mean ± SD (n = 3; * *p* < 0.05, ** *p* < 0.01, and *** *p* < 0.001 vs. control group); (**e**) CCK-8 analysis of Gel/SA-TA hydrogel with different TA concentrations (30 μg/mL, 60 μg/mL, and 120 μg/mL) in co-culture with 293T cells at different concentrations.

**Analysis of shape memory performance:** As shown in Figure 5a, the strip samples were immersed in 50 °C hot water for 60 s to open the triple helix structure of the gel and make it soft. Then, they were immersed in 25 °C water for 30 s to fix the temporary shape of the gel system due to the formation of a gelatin triple helix. The original linear distance of the strip Gel/SA-TA sample during shape memory recovery and the ratio of the linear distance between the two ends of the sample, namely, D_1_/D, were used as the evaluation criteria for the shape memory performance of the hydrogel. As shown in Figure 5b, when the gel monomer concentration is 20 wt%, Gel/SA-TA hydrogel shows the best shape memory performance, and the shape recovery rate reaches 74.85% ± 4.776% within eight minutes. This is because gel chains can spontaneously form reversible physical crosslinks when cooled (mainly triple helix structures). Increasing gel concentration means more gelatin chains per unit volume and a significantly higher density of physical crosslinking points formed. These physical crosslinks are the key to fixing the gel’s temporary shape. The higher density provides greater network locking, making the deformed shape more stable. Secondly, TA is rich in phenolic hydroxyl groups, which can form multiple dynamic hydrogen bonds with the amino, carboxyl, and hydroxyl groups on the gel chain, hydroxyl group, and carboxyl group on the SA chain. Increasing the gel concentration not only provides more sites that can bind to TA but also increases the hydrogen bonding interaction between gel chains. This significantly increases the dynamic physical crosslink density across the network, particularly, hydrogen bonding. Under stimuli, these dynamic bonds are more likely to break and reorganize, allowing the network to return to its permanent shape (dominated by covalent crosslinks) more efficiently.

**Analysis of biocompatibility:** As shown in Figure 5c, 293T cells had higher cell density and fewer clumps and dead cells on the Gel/SA-TA hydrogel with TA concentrations of 5 wt% and 6 wt%. The cell state was also significantly better than that of the blank control group and the Gel/SA-TA hydrogel with a TA concentration of 7 wt%. At the same time, the 293T cells showed a three-dimensional extension state under the inverted microscope, indicating that they had good growth and diffusion behavior on the Gel/SA-TA hydrogel.

The effect of Gel/SA-TA hydrogel on the activity of 293T cells is shown in Figure 5d. Their survival rate was higher than 90% in hydrogels at all TA concentrations. This indicated that the hydrogel did not produce any toxicity to 293T cells. Given that gel has excellent cytocompatibility and low antigenicity, has a positive effect on cell growth and adhesion, and is the main component of hydrogel, the inherent biocompatibility can be verified. In addition, a CCK-8 assay was used to evaluate the proliferation of 293T cells co-cultured with different TA concentrations for 72 h, as shown in Figure 5e. All hydrogels showed excellent proliferation, especially the cells separately cultured on Gel/SA-TA7 hydrogel at concentrations of 12 μg/mL and 120 μg/mL for 24 h. The relative proliferation rates at 48 h and 72 h were 108.485% ± 4.878%, 111.171% ± 3.421%, and 112.017% ± 2.4532%.

Based on the consideration and analysis of these performance characterization results, to complete the preparation of Gel/ SA-TA hydrogel, in this section, the gel concentration is set as 20 wt%, SA concentration as 3 wt%, TA concentrations as 5 wt% and 6 wt%, and the crosslinking time of CaCl_2_ solution as 30 min.

### 3.3. Performance of Gel/SA-TA@Fe^3+^ Bioprinting Ink

**Figure 6:**
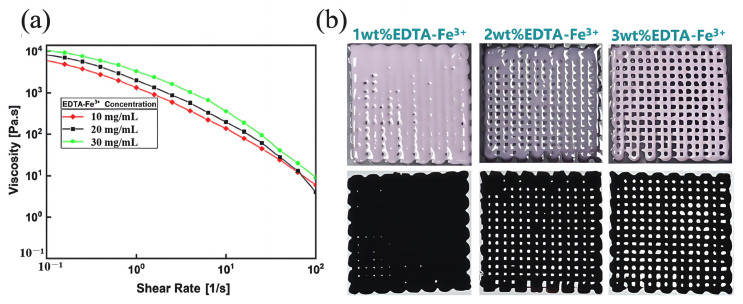
Characterization of DIW 3D printing performance of Gel/SA-TA@Fe^3+^ biological ink. (**a**) Rheological properties of Gel/SA-TA@Fe^3+^ biological inks with different Fe^3+^ concentrations at room temperature (25 °C); (**b**) printed renderings of Gel/SA-TA@Fe^3+^ bioink with different Fe^3+^ concentrations at 37 °C.

**Analysis of 3D printing performance characterization results:** As shown in Figure 6a, when the shear rate increased from 0.1 to 100 s^−1^, the viscosity of each Gel/SA-TA@Fe^3+^ bioink decreased below 100 Pa·s, indicating excellent shear-thinning behavior. Simultaneously, as the Fe^3+^ concentration rose from 1 wt% to 3 wt%, the ink’s viscosity increased from 6184 Pa·s to approximately 1 × 10^4^ Pa·s due to the strong metal coordination between TA and Fe^3+^. High viscosity is the key to maintaining 3D structural fidelity during DIW printing. Considering ink uniformity and structural stability, the printer barrel temperature was adjusted to 37 °C for hot extrusion to ensure efficient 3D printing. Figure 6b shows that this bioink exhibited optimal DIW printing performance at a 3 wt% Fe^3+^ concentration.

**Figure 7:**
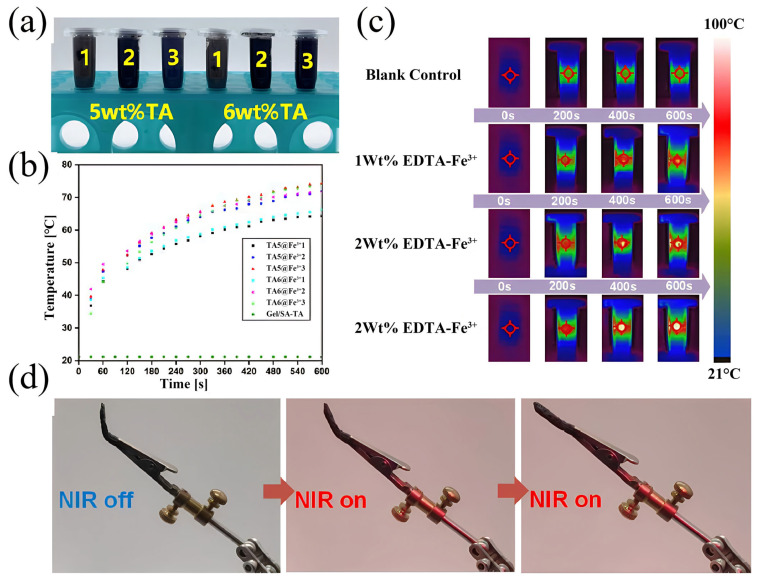
Characterization of photothermal properties of Gel/SA-TA@Fe^3+^ hydrogel. (**a**) Gel/SA-TA@Fe^3+^ biological inks with Fe^3+^ concentrations of 1 wt%, 2 wt%, and 3 wt%, from left to right; (**b**,**c**) temperature changes in Gel/SA-TA@Fe^3+^ biological inks with different TA and Fe^3+^ concentration ratios under NIR irradiation for 0~600 s; (**d**) shape memory recovery process of Gel/SA-TA@Fe^3+^ hydrogel under NIR irradiation.

**Analysis of photothermal properties:** Figure 7a shows Gel/SA-TA bioinks with 5 wt% and 6 wt% TA concentrations (n = 3). Figure 7b,c depict the relationship between bioink temperature and NIR irradiation time. They indicate that the Gel/SA-TA bioink showed no significant temperature change when irradiated at 808 nm (0.2 W/cm^2^) for 600 s at 20 °C. By contrast, the Gel/SA-TA@Fe^3+^ ink containing 3wt% EDTA-Fe^3+^ reached 74.3 °C under identical conditions. Of these, the bioink with 5wt% TA exhibited a more pronounced temperature increase than that with 6wt% TA under NIR irradiation. This demonstrates the hydrogel’s NIR stimulus-responsive behavior, with its temperature change primarily dependent on the Fe^3+^ concentration in TA@Fe^3+^ but largely unaffected by TA concentrations. Figure 7d and Figure S2 show the shape memory recovery process of this hydrogel under NIR irradiation.

In the Gel/SA-TA@Fe^3+^ hydrogel system, the ionic crosslink of SA is a rigid skeleton, the crystalline region of the gel provides the stationary phase, and the dynamic coordination bond of the TA-Fe^3+^ is the reversible phase. Under near-infrared light irradiation, TA absorbs light energy and converts it into heat energy, and local heating achieves two key functions: one is to break the TA-Fe^3+^ coordination bond to soften the network, and the other is to unspin the helix structure of the gel. At this point, the external force can easily deform. The temperature drops after removing the light, the TA-Fe^3+^ coordination bond reorganizes, and the gel re-forms the crystalline region, thereby locking its temporary shape. When irradiated with NIR again, the same thermal effect returns the material to the initial crosslinked state, completing the shape recovery.

### 3.4. Performance of Gel/SA-TA@Fe^3+^ Bioink Printing Samples

**Figure 8:**
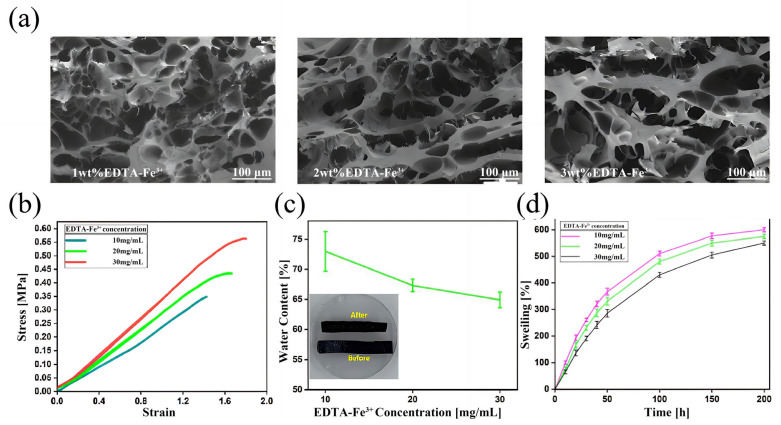
The physical properties of Gel/SA-TA bioink printing samples. (**a**) Electron microscopic scan of Gel/SA-TA@Fe^3+^ hydrogel morphology at different Fe^3+^ concentrations; (**b**) Gel/SA-TA@Fe^3+^ hydrogel tensile curves at different Fe^3+^ concentrations; (**c**) water content curves of Gel/SA-TA@Fe^3+^ hydrogel in CaCl_2_ solution after crosslinking for 30 min at different Fe^3+^ concentrations; (**d**) swelling rate curves of Gel/SA-TA@Fe^3+^ hydrogel at different Fe^3+^ concentrations. All experiments were performed in triplicate, and data are reported as mean ± SD (n = 3).

**Analysis of microscopic morphology:** As shown in Figure 8a, the Gel/SA-TA@Fe^3+^ hydrogel exhibited a loose interconnected porous structure across different Fe^3+^ concentrations. Specifically, samples with 1wt% Fe^3+^ formed large microscopic pores, averaging between 34 and 104 μm. As the Fe^3+^ concentration increased, the microstructure became more compact, significantly reducing pore number and size, reaching a minimum of about 13 μm. This indicates that the hydrogel developed higher crosslinking density due to coordination bonds between TA molecules and EDTA-Fe^3+^.

**Analysis of Mechanical properties:** As shown in Figure 8b, the average tensile modulus of the Gel/SA-TA@Fe^3+^ hydrogel increased from 0.30763 ± 0.019 MPa to 0.35881 ± 0.021 MPa as the Fe^3+^ concentration rose from 1 wt% to 3 wt%. This enhancement is due to the increasing number of coordination bonds in TA@Fe^3+^ with a higher Fe^3+^ concentration. At the 3 wt% Fe^3+^ level, the average modulus reached its peak value at 0.562 ± 0.0286 MPa, representing a 1.4-fold increase over the Gel/SA-TA hydrogel and exceeding values reported in previous studies [58,59]. Similarly, its average elastic modulus also strengthened with increasing Fe^3+^ content.

**Analysis of water content performance:** Figure 8c shows that the water content of the Gel/SA-TA@Fe^3+^ hydrogel decreases with increasing Fe^3+^ concentrations. This indicates its internal structure becomes denser as the density of TA@Fe^3+^ complexation products increases. At 3 wt% Fe^3+^, the hydrogel reached its lowest water content (64.86% ± 1.28%). Figure 8d compares the hydrogel before and after water loss. The swelling curve under different Fe^3+^ concentrations after 30 min of crosslinking is shown in Figure 8d. Each group reached near-swelling equilibrium after 200 h of immersion in PBS. Furthermore, swelling performance decreased with higher Fe^3+^ concentrations, consistent with the water content results. The highest swelling degree (600.65% ± 8.2%) occurred at 1 wt% Fe^3+^.

**Figure 9:**
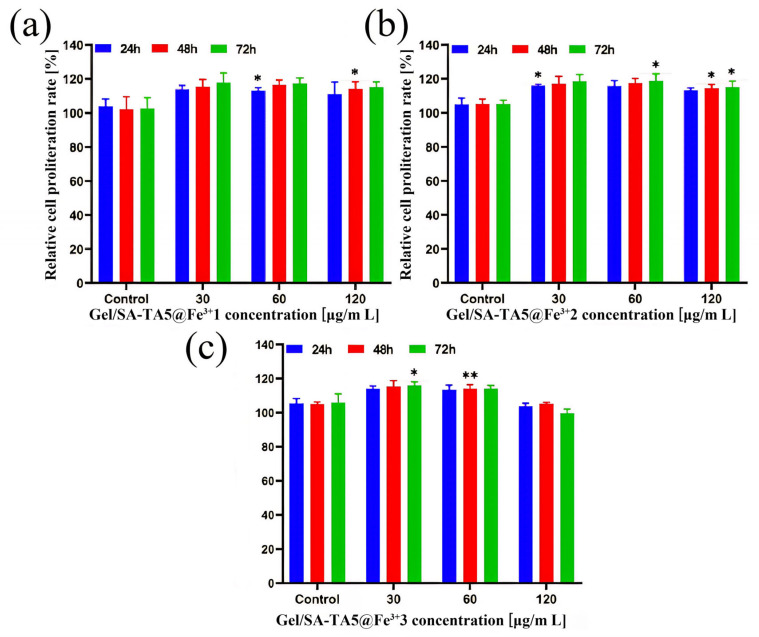
Quantitative analysis of the effects of Gel/SA-TA@Fe^3+^ hydrogel on 293T cell proliferation; (**a**) Gel/SA-TA@1wt%Fe^3+^; (**b**) Gel/SA-TA@2wt%Fe^3+^; (**c**) Gel/SA-TA@3wt%Fe^3+^. Data are reported as mean ± SD (n = 3; * *p* < 0.05, ** *p* < 0.01, vs. negative control group).

**Analysis of Biocompatibility:** EDTA-FeNa is an iron compound used as an oral supplement for iron deficiency, and it exhibits good biocompatibility and biomedical potential. Figure 9a–c show a 293T cell proliferation quantitative analysis of the Gel/SA-TA@Fe^3+^ hydrogel at different Fe^3+^ concentrations (1 wt%, 2 wt%, and 3 wt%). The results indicate that while the relative proliferation rate at a 120 μg/mL hydrogel concentration was lower than at 30 μg/mL and 60 μg/mL, cells co-cultured with this hydrogel maintained significant proliferation across all EDTA-Fe^3+^ concentrations. Specifically, the 3 wt% EDTA-Fe^3+^ hydrogel at 120 μg/mL showed a slight decrease in relative proliferation after 72 h of co-culturing. Nevertheless, its proliferation rates reached 103.629% ± 3.268% (24 h), 105.231% ± 2.021% (48 h), and 99.733% ± 3.853% (72 h). These results indicate that Gel/SA-TA@Fe^3+^ hydrogel is less cytotoxic.

**Figure 10:**
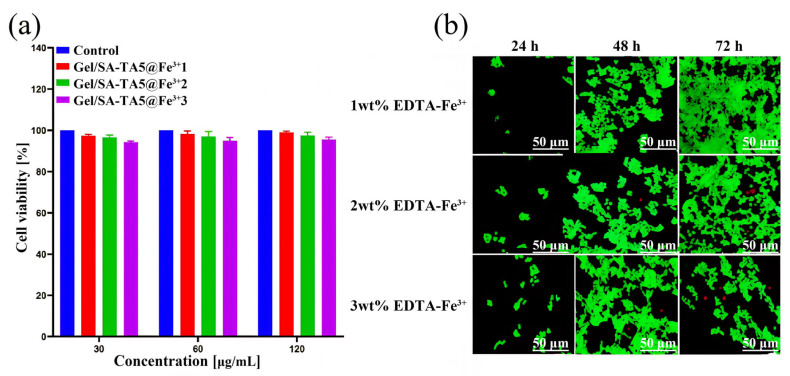
Effects of Gel/SA-TA@Fe^3+^ hydrogel on 293T cell activity. (**a**) Quantitative analysis diagram of cell activity; (**b**) fluorescence staining of live/dead cells (green fluorescence represents live cells; red fluorescence represents dead cells). Data are reported as mean ± SD (n = 3).

Figure 10a,b demonstrate good 293T cell viability in the Gel/SA-TA@Fe^3+^ hydrogel across different Fe^3+^ concentrations (1–3 wt%). From 24 h to 72 h of co-culturing, cells proliferated significantly with minimal death, showing sustained growth. Viability analysis at 72 h revealed the highest activity in the 1 wt% EDTA-Fe^3+^ samples and the lowest in the 3 wt% formulations. Critically, cell survival exceeded 90% in all concentrations, confirming the hydrogel’s minimal cytotoxicity toward 293T cells.

Based on the analysis of the above performance characterization results, the TA concentration was set as 5 wt% and the EDTA-Fe^3+^ concentration as 3 wt%. The optimal concentration of the above components will be used to complete the study of Gel/SA-TA@Fe^3+^ hydrogel for use in wearable flexible sensors.

### 3.5. Analysis of Gel/SA-TA@Fe^3+^ Impedance Feedback Signals in Response to Deformable and Infrared Stimuli

**Figure 11:**
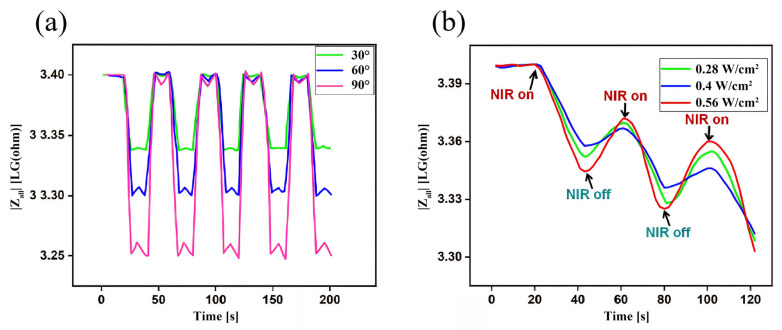
Impedance changes in Gel/SA-TA@Fe^3+^ hydrogel in response to external stimuli. (**a**) Impedance change in Gel/SA-TA@Fe^3+^ hydrogel during finger bending; (**b**) impedance changes in Gel/SA-TA@Fe^3+^ hydrogel under NIR irradiation.

**Impedance feedback signal analysis in response to deformation stimulation:** As shown in Figure 11a, when the finger-bending angle changes, the impedance value of the Gel/SA-TA@Fe^3+^ hydrogel increases significantly. When the finger angle returns to the initial state, the impedance value of the material will show a downward trend, and the degree of the impedance change trend in the hydrogel will increase with the increased bending angle. This is due to the flexible characteristics of hydrogels, which easily undergo shape changes under small external forces, changing their resistance due to the alteration of their internal free ion concentrations and movement directions. This is a transition from a mechanical to an electrical signal. Moreover, we used Arduino UNO to measure the impedance of the hydrogel; the experimental procedure and results are shown in Figure S3. The impedance spectrum of the Gel/SA-TA@Fe^3+^ hydrogel at 1 Hz to 100 kHz is shown in Figure S4.

**Impedance feedback signal analysis in response to infrared stimulation:** As shown in Figure 11b, at the moment of each instance of NIR irradiation, the impedance value of the Gel/SA-TA@Fe^3+^ hydrogel showed a significant downward trend. When the NIR irradiation was turned off, the impedance value showed an upward trend, and the degree of impedance change trend increased with the increase in NIR intensity.

## 4. Conclusions

In this study, two stimulus-responsive hydrogels were designed using Gel, SA, TA, and EDTA-FeNa materials. They can be applied to DIW printing with good performance. Thus, according to multiple stimulus–response tests, the hydrogel can be used for DIW 3D printing. The application of wearable flexible sensors was also studied. The main conclusions are as follows:

1. TA introduces shear-thinning characteristics to the hydrogel system during viscosity changes, improving the preparation efficiency while ensuring the accuracy of the printed Gel/SA-TA 3D models. Furthermore, TA’s ability to generate intermolecular forces with the Gel/SA matrix results in a 6.6-fold increase in tensile modulus compared with pure Gel/SA hydrogel. The resulting composite also demonstrates good cytocompatibility and a positive effect on 293T cell proliferation.
2. Adding Fe^3+^ provides the Gel/SA-TA hydrogel system with a dual stimulus–response to NIR and temperature. Given the strong metal coordination between TA and Fe^3+^, the tensile strength of the Gel/SA-TA@Fe^3+^ hydrogel is also significantly improved compared with the Gel/SA-TA hydrogel. Meanwhile, the Gel/SA-TA@Fe^3+^ hydrogel also exhibits good cytocompatibility.
3. The sensing mechanism of the hydrogels was explored, and the impedance of the Gel/SA-TA@Fe^3+^ hydrogel was smaller and more stable at a high frequency. When the hydrogel is actually used as a flexible sensor, the working voltage value should be set at 0.02 V. Finally, the Gel/SA-TA@Fe^3+^ hydrogel had good NIR stimulus–response performance and deformation–response performance, which is of great significance for the real-time monitoring of vital signs and human movement in the daily use of hydrogels in biomedical devices.
4. In future studies, more comprehensive mechanical property tests (such as compression, bending, robustness, and durability tests) and more extensive primary cell experiments (such as other primary cells and stem cell experiments) will enhance the exhaustiveness of this research.

## Figures and Tables

**Table 1:** Ratio table for each group of Gel/SA-TA biological inks.

No.	SA (wt%)	Gel (wt%)	TA (wt%)
1	3	5	\
2	3	10	\
3	3	15	\
4	3	20	1
5	3	20	3
6	3	20	5
7	3	20	6
8	3	20	7
9	3	20	9

**Table 2:** Ratio table for each group of Gel/SA-TA@Fe^3+^ biological inks.

No.	SA (wt%)	Gel (wt%)	TA (wt%)	EDTA-FeNa (wt%)
1	3	20	5	1
2	3	20	5	2
3	3	20	5	3
4	3	20	6	1
5	3	20	6	2
6	3	20	6	3

## Data Availability

All data needed to evaluate the conclusions of this study are present in the paper and/or the Supplementary Materials. Additional data related to this study may be requested from the authors.

## Supplementary Materials

The following supporting information can be downloaded at https://www.mdpi.com/article/10.3390/mi16070788/s1: Figure S1: Composition of DIW 3D printer. Figure S2: Deformation of Gel/SA-TA@Fe^3+^ bioink under NIR irradiation. Figure S3: Characterization of electrical conductivity of Gel/SA-TA@Fe^3+^ hydrogels: (a) physical drawing of the Arduino UNO impedance monitoring system; (b) implementation of impedance monitoring. Figure S4: Impedance spectrum of Gel/SA-TA@Fe^3+^ hydrogel at 1 Hz to 100 kHz.

## Abbreviations

The following abbreviations are used in this manuscript:

DIW	Direct ink writing
AM	Additive manufacturing
SA	Sodium alginate
TA	Tannic acid
NIR	Near-infrared
EDTA	Ethylene diamine tetra acetic acid
