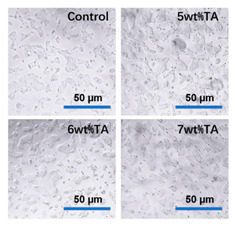# Correction: Wu et al. 3D-Printed Multi-Stimulus-Responsive Hydrogels: Fabrication and Characterization. *Micromachines* 2025, *16*, 788

**DOI:** 10.3390/mi17010098

**Published:** 2026-01-12

**Authors:** Jinzhe Wu, Zhiyuan Ma, Qianqian Tang, Runhuai Yang

**Affiliations:** 1 School of Electronic Engineering, Naval University of Engineering, Wuhan 430033, China; wujinzhe@ustc.edu (J.W.); zhiyuan_ma@nue.edu.cn (Z.M.); 2 Chaohu Clinical Medical College, Anhui Medical University, Chaohu 238000, China; qqt19960806@163.com; 3 The Chaohu Hospital of Anhui Medical University, Chaohu 238000, China

## Error in Figure

In the original publication [1], there was a mistake in Figure 5c “inverted microscope image of 293T cells after Gel/SA-TA hydrogel coculture for 72 h” as published. During the figure insertion process, an incorrect, though visually similar, figure was inadvertently placed in this position.

The corrected Figure 5c “inverted microscope image of 293T cells after Gel/SA-TA hydrogel coculture for 72 h” appears below. The authors state that the scientific conclusions are unaffected. This correction was approved by the Academic Editor. The original publication has also been updated.