# Supplementary figures and images for: 3D-Printed Multi-Stimulus-Responsive Hydrogels: Fabrication and Characterization
Source: Micromachines (Basel). 2025 Jul 1;16(7):788. doi: 10.3390/mi16070788 (PMC12298978; doi:10.3390/mi16070788)

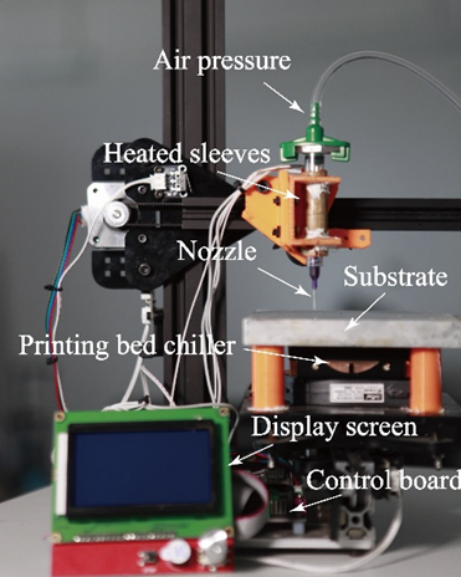

Supplement: Supplementary file 1 [file micromachines-16-00788-s001.zip › FigureS1.png]

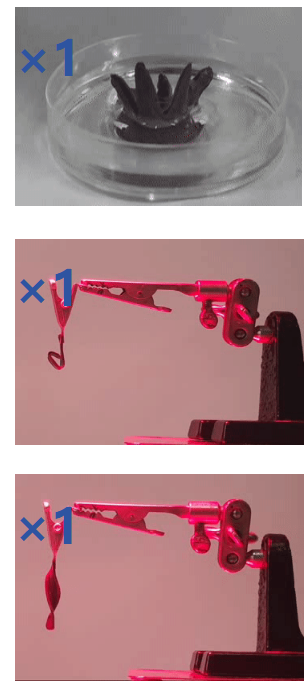

Supplement: Supplementary file 1 [file micromachines-16-00788-s001.zip › FigureS2.png]

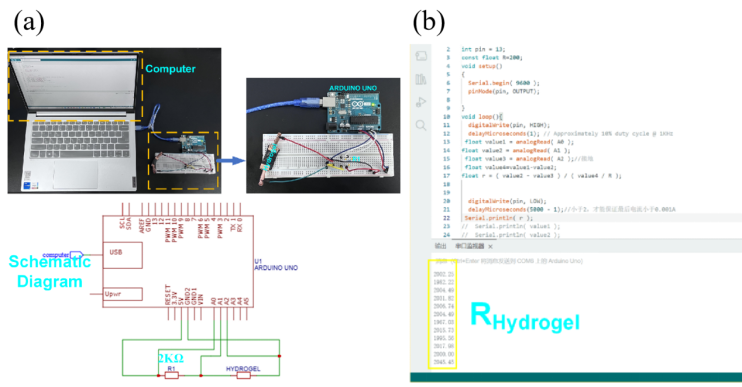

Supplement: Supplementary file 1 [file micromachines-16-00788-s001.zip › FigureS3.png]

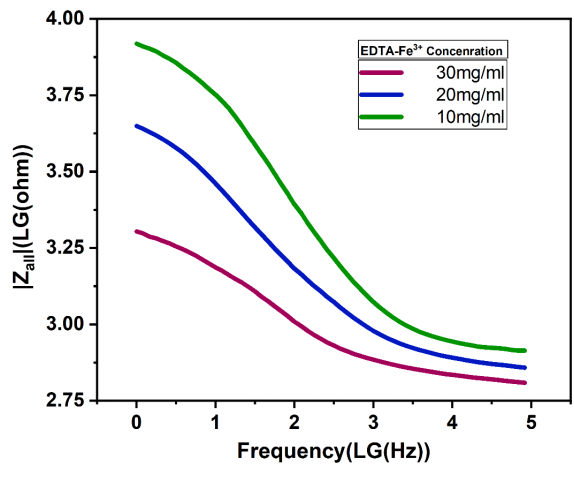

Supplement: Supplementary file 1 [file micromachines-16-00788-s001.zip › FigureS4.png]
